# Draft Genome Sequences of Pseudomonas koreensis Strain UASWS1668, Bacillus megaterium Strain UASWS1667, and *Paenibacillus* sp. Strain UASWS1643, Considered Potential Plant Growth-Promoting Rhizobacteria

**DOI:** 10.1128/MRA.00768-20

**Published:** 2020-08-13

**Authors:** Julien Crovadore, Bastien Cochard, Romain Chablais, Martine Haenzi, François Raffini, François Lefort

**Affiliations:** a Plants and Pathogens Group, Research Institute Land Nature and Environment, Geneva School of Engineering, Landscape and Architecture (HEPIA), HES-SO University of Applied Sciences and Arts Western Switzerland, Jussy, Geneva, Switzerland; University of Southern California

## Abstract

Plant growth-promoting rhizobacteria (PGPR) include species in the genera *Bacillus*, *Paenibacillus*, and *Pseudomonas*. We report here the draft genome sequences of the strains Pseudomonas koreensis UASWS1668 and Bacillus megaterium UASWS1667, isolated from a horse chestnut tree, and *Paenibacillus* sp. strain UASWS1643, isolated from a tomato stem. Auxin production and phosphate solubilization were biochemically confirmed.

## ANNOUNCEMENT

*Pseudomonas koreensis* strain UASWS1668 and Bacillus megaterium strain UASWS1667, isolated from a horse chestnut tree, and *Paenibacillus* sp. strain UASWS1643, isolated from a tomato stem, were identified by 16S rRNA gene sequencing and later confirmed by whole 16S rRNA gene sequences extracted from their genome assemblies. A whole-genome sequencing strategy was chosen to confirm the potential of these bacteria as biostimulants. *Paenibacillus* sp. strain UASWS1643 showed 97.52% shared identity with the closest NCBI-referenced strain, Paenibacillus xylanexedens strain PAMC 22703 (1). *P. koreensis* has shown antifungal activities and plant growth-promoting rhizobacterium (PGPR) properties (2), particularly in heavy metal-contaminated soils (3) and under high-salt conditions (4–6), increasing plant fresh weight and root development. Bacillus megaterium is known for plant growth induction (7, 8), its antifungal properties, its resistance to heavy metals (8, 9), and adaption to acidic stress (10). The genus *Paenibacillus*, widely present in the soils and rhizospheres of many plants, commonly hosts PGPR species (11, 12), with nitrogen fixation, phosphate solubilization, auxin production, and siderophore secretion ability (11, 12).

Bacteria were isolated from the diseased tissues of horse chestnut or tomato according to a published method (13). DNA was extracted from pure cultures grown exponentially from a single colony in LB broth, with a modified cetyltrimethylammonium bromide protocol (14). Sequenced 16S amplicons produced with primers 27F and 1482 R (15) were identified by BLAST (16). Sequencing libraries were produced with the TruSeq Nano DNA library preparation kit (Illumina, USA). Whole-genome sequencing was performed with a MiniSeq high-output kit, in one Illumina MiniSeq run in 2 × 151-bp paired-end read format. The read quality was controlled with FastQC version 0.11.5 (17). Genome assemblies were computed with the SPAdes version 3.13.0 genome assembler (18), set to “paired-end” assembly and “careful” mode. The resulting contigs were ordered with BioEdit version 7.0.5 (19) and analyzed with QUAST version 4.6.3 (20), with the setting “QUAST: skip contigs shorter than 200 bp.” Automated gene annotations were carried out by the Prokaryotic Genome Annotation Pipeline (PGAP) version 4.1 (21) and RAST version 2.0 (22), with the ClassicRAST annotation scheme. Indole-3-acetic acid (IAA) production and phosphate solubilization were estimated according to published protocols (23, 24).

The sequence details are given in Table 1. Using PlasmidFinder version 1.3 (25) and plasmidSPAdes (26), with default settings, no plasmids were found in Pseudomonas koreensis UASWS1668 or *Paenibacillus* sp. strain UASWS1643, while Bacillus megaterium UASWS1667 displayed 7 plasmids. No complete transposons or phages were found in Bacillus megaterium UASWS1667, whereas *Paenibacillus* sp. strain UASWS1643 harbored 1 phage on contig 116, and Pseudomonas koreensis UASWS1668 had 2 phages in contigs 68 and 70. Toxins and superantigens as well as virulence and disease genes were absent from all 3 strains, allowing these bacteria to be considered for agronomical use. All strains were predicted to synthesize antibiotics of the bacitracin type and to resist antibiotics and heavy metals, with 54 genes for Pseudomonas koreensis UASWS1668, 37 genes for Bacillus megaterium UASWS1667, and 47 genes for *Paenibacillus* sp. strain UASWS1643. Biochemical tests confirmed that the three strains produce auxin and solubilize phosphate, as predicted by the genome sequences.

**TABLE 1:** Genome sequence statistics summary and characteristics for Pseudomonas koreensis strain UASWS1668, Bacillus megaterium strain UASWS1667, and *Paenibacillus* sp. strain UASWS1643

Characteristic	Data for strain:		
	*Paenibacillus* sp. UASWS1643	Bacillus megaterium UASWS1667	Pseudomonas koreensis UASWS1668
Total length (bp)	7,354,805	5,171,380	6,177,111
GC content (%)	45.88	38.04	60.27
No. of CDSs^*a*^ (PGAP)	6,422	5,232	5,660
No. of CDSs (RAST)	6,887	5,600	5,440
No. of tRNAs	63	70	59
No. of rRNAs	18	13	6
No. of phages	1	0	2
No. of plasmids	0	7	0
No. of auxin genes	4	4	4
No. of phosphorus metabolism genes	52	26	38
No. of nitrogen metabolism genes	34	12	14
Sequencing yield (Mbp)	445.96	550.32	574.37
Final coverage (×)	60.6	106.4	93
SRA accession no. for raw reads	SRX6863606	SRX6863642	SRX6863625
No. of reads	2,953,376	3,644,512	3,803,796
No. of scaffolds	117	113	70
Scaffold *N*_50_ (bp)	253,767	1,095,828	663,749
GenBank accession no. for assembled genome	VXKZ01000000	VXLA01000000	VXLB01000000
BioProject accession no.	PRJNA543413	PRJNA543415	PRJNA543411
BioSample accession no.	SAMN11658785	SAMN11658787	SAMN11658786

a CDSs, coding DNA sequences.

### Data availability.

This genome sequencing project has been deposited in the NCBI Sequence Read Archive (SRA) and genome databases. The genome, SRA, BioProject, and BioSample accession numbers and statistics for the three individual strains are given in Table 1.
